# Associated Factors Relating to the Success of Labor Induction among Pregnant Women Diagnosed with Fetal Intrauterine Growth Restriction: A Retrospective Cohort Study from France

**DOI:** 10.1055/a-2764-2369

**Published:** 2026-01-16

**Authors:** Phuc Nhon Nguyen, Anna Ramos

**Affiliations:** 1 Service de Gynécologie-Obstétrique, Maternité, Chirurgie Gynécologique, Centre Hospitalier Universitaire d'Orléans, Orléans, France; 2 Faculté de Médecine de Tours, Université de Tours, Tours, France

**Keywords:** Doppler ultrasound, induction of labor, fetal growth restriction, gestational age, vaginal birth

## Abstract

**Objective:**

This study aimed to identify factors related to the successful induction of labor (IOL) among pregnancies complicated with fetal growth restriction (FGR).

**Methods:**

We performed a retrospective cohort study between January and December 2024 at Orléans University Hospital, Orléans, France. The study enrolled all singleton pregnancies complicated by FGR and underwent IOL. Logistic regression was used to reveal the associated factors relating to the success of IOL, following two criteria, including Bishop score after IOL greater than 7 points (criterion 1) and vaginal birth (criterion 2).

**Results:**

Bishop score had a negative correlation with IOL duration. This study did not find statistically significant clinical factors related to the success of the Bishop score change of more than 7 points. However, absence of Doppler abnormalities and IOL at term, more than 37 weeks, were related to the success rate of vaginal delivery (OR [95% CI]: 22.5 [3.240–156.269] and OR [95% CI]: 22.5 [3.240–156.269], respectively; *p* < 0.05).

**Conclusion:**

Bishop score before IOL helps in reducing the duration of IOL. In FGR pregnancies with a normal Doppler ultrasound scan and gestational age at IOL greater than 37 weeks, these are good prognostic factors for vaginal delivery; however, further research is needed to explore more associated factors.

## Introduction

Fetal growth restriction (FGR) is often defined as an estimated fetal weight (EFW; grams) less than the 10th percentile for gestational age by prenatal ultrasound evaluation. 1 FGR is described with an incidence of 5% to 10%, leading to a significant risk of perinatal mortality, neonatal morbidity, and long-term health defects. 2 Therefore, termination of pregnancy at an appropriate gestation, balancing the risk and neonatal outcomes, is recommended. 3 4 Induction of labor (IOL) is a common procedure without adverse materno–fetal outcomes. 5 IOL in FGR pregnancy did not increase the rate of cesarean delivery. However, pregnant women with a diagnosis of FGR are significantly more likely to have a cesarean delivery for non-reassuring fetal heart tracing than a cesarean delivery for other indications (Adjusted odds ratio (AOR): 3.08; 95% CI: 1.13–8.43) or vaginal birth (AOR: 4.0; 95% CI: 1.97–8.08) in the late preterm period. 6

Until today, associated factors related to the success of IOL have not been well demonstrated. In general population, several factors relating to success rate of IOL have been known well such as age, primiparity, method of conception, birth weight, hypertensive disorders of pregnancy, and simplified Bishop score should be considered the predictor of failed IOL. 7 8 9 Alayu et al have found that mid–upper arm circumstance 23 to 28 cm (AOR = 2.55, 95% CI: 1.19–5.47), multiparity (AOR = 3.01, 95% CI: 1.430–6.33), favorable Bishop (AOR = 3.79, 95% CI: 1.74–8.26), oxytocin with cervical ripening method (AOR = 3.74, 95% CI: 1.99–7.04), and birth weight less than 4,000 g (AOR = 5.40, 95% CI: 1.54–18.91) were factors significantly associated with successful vaginal delivery following induction. 10 However, in pregnancies complicated with FGR, the prognostic factors remain variable. According to Horowitz and Feldman, pregnant women with oligohydramnios and those requiring prostaglandins for cervical ripening should be counseled regarding a significantly higher risk of cesarean delivery. 11

This study aims to investigate the factors relating to the successful rate of IOL in pregnancies complicated by FGR at our maternity unit of Orleans University Hospital.

## Materials and Methods

**Fig. 1:**
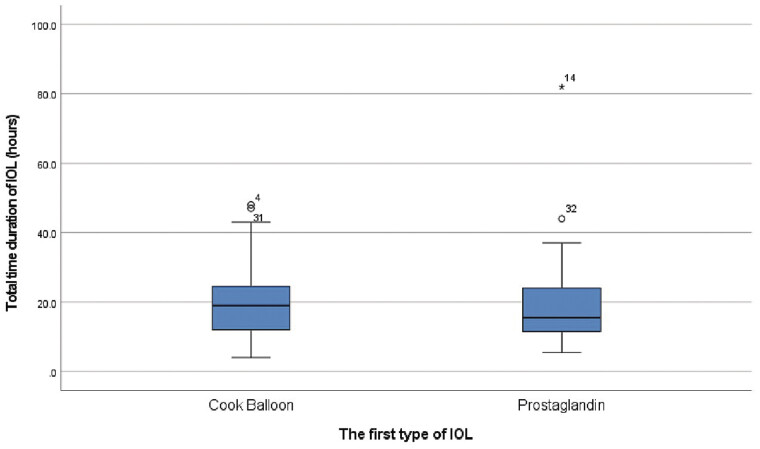
Duration of IOL (hours) following the methods of IOL. IOL, induction of labor.

This retrospective cohort study analyzed the records of patients who gave birth in the Maternity Department of the Orléans University Hospital, Orleans, France, between January 1, 2024, and December 31, 2024 ( Fig. 1 ). This study was accepted by the Clinical Research Room of the institution with the ethical code: 2025-HORS-RIPH-01.

Inclusion criteria included a pregnancy diagnosed with FGR from 32 weeks of amenorrhea and an unfavorable cervix (Bishop score ≤6). Inclusion criteria were pregnancies with a non-anomalous, vertex, singleton gestation and complicated by late FGR (gestational age ≥32 weeks of gestation at diagnosis), as defined by the proposed Delphi consensus criteria, including

- Two solitary parameters: Abdominal circumference (AC) and/or EFW ratio <3rd centile.
- Or at least two out of three of the following: (a) AC/EFW <10th centile; (b) AC/EFW crossing centiles >2 quartiles on growth centiles; (c) cerebroplacental ratio <5th centile or uterine artery pulsatility index (UA-PI) >95th centile. 3 12

Non-inclusion criteria were twin pregnancy, breech presentation, scarred uterus, fetal death in utero, cesarean section (CS) before labor, fetus with morphological anomalies, and labor induction by combined cervical ripening at the same time.

IOL methods included mechanical methods (Cook® balloon) and prostaglandins (Propess®, Angusta®). In case the patient was induced by two or three methods, the first method of IOL was used for the evaluation of criterion 1.

### Collection of Variables

The variables concerning materno–fetal and pregnancy characteristics were collected and classified as

- Continuous variables: Maternal age (years), body mass index (BMI: kg/m ^2^ ), EFW (grams), gestational age at diagnosis of FGR and at IOL (weeks), and initial Bishop score (points).
- Categorical variables: Smoking, parity, amount of amniotic fluid, fetal Doppler indices, and need for amniotomy/oxytocin.

### Statistical Analysis

Statistical analyses were performed using the Statistical Package for the Social Sciences (SPSS) version 27.0 (IBM Corp., Armonk, NY). The analysis was performed using all available data. The distribution of continuous variables was explored using histograms, skewness, and kurtosis to identify a normal distribution. Descriptive statistics were expressed as mean and standard deviation (mean ± SD) for quantitative, percentage, and interquartile range for continuous variables, depending on the distribution of data. The independent sample *t* -test and the Mann–Whitney U test were used to compare continuous variables following the distribution of the data. Frequency data with percentage and comparison of categorical variables were performed across categories using the χ ^2^ (chi-square) test. If the counting variable has a theoretical number < 5 in each cell (>25% of the table), the *p* -value is obtained by Fisher's exact probability test.

The odds ratio (OR) was calculated from the 2 × 2 table. Indicators and effect values were expressed with a 95% confidence interval (CI). To determine whether there was an association between cervical ripening method and Bishop's change success ≥7 and mode of delivery (Vaginal birth), univariate and multivariate logistic regression were performed using gestational age and newborn weight as the adjustment variables. Test results were considered significant at a *p* -value of <0.05.

## Results

The materno–fetal characteristics were not significantly different between the two groups. IOL using the Cook Balloon required more oxytocin than that of Prostaglandin E (PGE). Indeed, the rate of oxytocin use was higher in the mechanical group than in the PGE group (73.9% vs. 26.1%, *p* < 0.05; Table 1 ). The duration of IOL (hours) following the methods of IOL was not different between the two groups ( Fig. 1 ).

**Table 1:** Maternal characteristics in terms of induction of labor methods in the present study

Characteristics		Overall ( *N* = 47)	Balloon ( *N* = 27)	Prostaglandin ( *N* = 20)	*p* -Value
Maternal age (years)	<20	1 (100)	0 (0.0)	1 (5.0)	0.675 b
	20–34	38 (100.0)	22 (57.9)	16 (42.1)	
	≥35	8 (100.0)	5 (62.5)	3 (37.5)	
Maternal height (cm)	Mean ± SD (min–max)	162.68 ± 6.55 (147–178)	163.11 ± 7.51 (147–178)	162.10 ± 5.12 (150–172)	0.606 a
Prepregnancy BMI (kg/m ^2^ )	Non-obese (BMI <30)	37 (100.0)	22 (59.5)	15 (40.5)	0.723 b
	Obese (BMI ≥30)	10 (100.0)	5 (50.0)	5 (50.0)	
BMI in pregnancy (kg/m ^2^ )	Non-obese (BMI <30)	32 (100.0)	21 (65.6)	11 (34.4)	0.122 d
	Obese (BMI ≥30)	15 (100.0)	6 (40.0)	9 (60.0)	
Smoking	No	34 (100.0)	21 (61.8)	13 (38.2)	0.511 d
	Yes	13 (100.0)	6 (46.2)	7 (53.8)	
Gravida	≥1	28 (100.0)	15 (53.6)	13 (46.4)	0.561 d
	0	19 (100.0)	12 (63.2)	7 (36.8)	
Parity	Multiparous	18 (100.0)	8 (44.4)	10 (55.6)	0.226 d
	Primiparous	29 (100.0)	19 (65.5)	10 (34.5)	
Amniotic fluid	Normal	37 (100.0)	20 (54.1)	17 (45.9)	0.481 b
	Abnormal	10 (100.0)	7 (70.0)	3 (30.0)	
Decreased fetal movement	Present	4 (100.0)	1 (25.0)	3 (75.0)	0.298 b
	Absent	43 (100.0)	26 (60.5)	17 (39.5)	
Doppler abnormalities e	Present	7 (100.0)	7 (100.0)	0 (0.0)	** 0.015 b**
	Absent	40 (100.0)	20 (50.0)	20 (50.0)	
Umbilical Doppler	Normal	43 (100.0)	23 (53.5)	20 (46.5)	0.126 b
	Abnormal ^f^	4 (100.0)	4 (100.0)	0 (0.0)	
Type of abnormal amniotic fluid	Oligohydramnios	9 (100.0)	6 (66.7)	3 (33.3)	1.0 b
	Amnios	1 (100.0)	0 (0.0)	1 (100.0)	
Gestational age at diagnosis (weeks)	<32	12 (100.0)	9 (75.0)	3 (25.0)	0.335 b
	32-37	32 (100.0)	16 (50.0)	16 (50.0)	
	≥37	3 (100.0)	2 (66.7)	1 (33.3)	
CTG before IOL	Normal	44	26 (59.1)	18 (40.9)	0.176 b
	Suspected	2 (100.0)	0 (0.0)	2 (100.0)	
	Pathologic	1 (100.0)	1 (100.0)	0 (0.0)	
Gestational age at IOL (weeks)	<37	7 (100.0)	6 (85.7)	1 (14.3)	0.213 b
	≥37	40 (100.0)	21 (52.5)	19 (47.5)	
Duration of IOL (hours)	Median [IQR: 25%–75%] (min–max)	12.0 [16.0–24.0] (4.0–82.0)	19.0 [12.0–25.0] (4.0–48.0)	15.5 [11.3–24.0] (5.5–82.0)	0.948 a
Newborn weight (grams)	<2,500	33 (100.0)	20 (60.6)	13 (39.4)	0.535 b
	≥2,500	14 (100.0)	7 (50.0)	7 (50.0)	
Bishop score before IOL (points)	3–6	25 (100.0)	11 (44.0)	14 (56.0)	0.076 b
	<3	22 (100.0)	16 (72.7)	6 (27.3)	
Need for oxytocin	Yes	23 (100.0)	17 (73.9)	6 (26.1)	** 0.025 b**
	No	23 (100.0)	10 (41.7)	14 (58.3)	
Duration of IOL (hours)	<24	31 (100.0)	18 (58.1)	13 (41.9)	1.0 b
	≥24	16 (100.0)	9 (56.3)	7 (43.8)	

Abbreviations: BMI, body mass index (kg/m ^2^ ); CTG, cardiotocography; IOL, induction of labor.

Data were presented as *n* (%), mean ± SD (min–max), median [IQR: 25%–75%].

Prostaglandin, including Dinoprostone (Propess) and Misoprostone (Angusta), and a combination of two methods of prostaglandin.

a Independent sample test.

b Fisher's exact test.

c Independent-sample Mann–Whitney U test.

d Chi-square test.

e Others: Systemic lupus erythematosus, gestational cholestasis.

f Reduction in end-diastolic flow (increasing resistance index values, pulsatility index values, and systolic/diastolic ratio and absent end-diastolic flow (AEDF).

In this study, maternal and fetal factors were not found to be related to the success of labor induction according to criterion 1 (Bishop score, after labor induction, with more than 7 points). Overall, 55.56% (15/27 cases) of patients in the Cook Balloon group had a Bishop score of ≥7 after cervical ripening, compared to 65.0% (13/20 cases) in the prostaglandin group. These results were not significantly different (OR: 0.673 [0.204–2.217], *p* = 0.514; Table 2 ). Table 3 shows that the absence of Doppler abnormalities and labor induction at term more than 37 weeks are related to the success rate of vaginal delivery (OR [95% CI]: 22.5 [3.240–156.269]; 22.5 [3.240–156.269], respectively; *p* < 0.05).

**Table 2:** Factors related to the success of induction of labor following criterion 1 (Bishop score ≥7 points)

Variable	Characteristics	Success ( *N* = 38)	Failed ( *N* = 9)	OR [95% CI]	*p* -Value
Maternal age (years)	<35	23 (82.1)	16 (84.2)	0.863 (0.180–4.134)	1.0 a
	≥35	5 (17.9)	3 (15.8)	Ref	
Maternal height (cm)	≥160	20 (71.4)	14 (73.7)	0.893 (0.241–3.308)	0.893 b
	<160	8 (28.6)	5 (26.3)	Ref	
Prepregnancy obesity (BMI ≥30 kg/m ^2^ )	Absent	21 (75.0)	16 (84.2)	0.563 (0.125–2.523)	0.718 a
	Present	7 (25.0)	3 (15.8)	Ref	
Obesity in pregnancy (BMI ≥30 kg/m ^2^ )	Absent	20 (71.4)	12 (63.2)	1.458 (0.421–5.047)	0.551 b
	Present	8 (28.6)	7 (36.8)	Ref	
Parity	Multiparous	12 (42.9)	6 (31.6)	1.625 (0.478–5.521)	0.435 b
	Primiparous	16 (57.1)	13 (68.4)	Ref	
Volume of amniotic fluid	Normal	23 (82.1)	14 (73.7)	1.643 (0.403–6.705)	0.496 a
	Abnormal (oligohydramnios)	5 (17.9)	5 (26.3)	Ref	
Decreased fetal movement	Absent	27 (96.4)	16 (84.2)	5.063 (0.485–52.878)	0.289 a
	Present	1 (3.6)	3 (15.8)	Ref	
Doppler abnormality	Absent	25 (89.3)	15 (78.9)	2.222 (0.436–11.320)	0.417 a
	Present	3 (10.7)	4 (21.1)	Ref	
Umbilical Doppler abnormality	Absent	27 (96.4)	16 (84.2)	5.063 (0.485-–2.878)	0.289 a
	Present	1 (3.6)	3 (15.8)		
CTG before IOL	Normal	25 (89.3)	19 (100.0)	NA	0.262 a
	Suspected/Abnormal	3 (10.7)	0 (0.0)		
Gestational age at IOL (weeks)	≥37	24 (85.7)	16 (84.2)	1.125 (0.221–5.714)	1.0 a
	<37	4 (14.3)	3 (15.8)	Ref	
Newborn weight (grams)	≥2,500	10 (35.7)	4 (21.1)	2.083 (0.542–8.011)	0.281 b
	<2,500	18 (64.3)	15 (78.9)	Ref	
Bishop score before IOL (points)	≥3	16 (57.1)	9 (47.4)	1.481 (0.459–4.778)	0.510 b
	<3	12 (42.9)	10 (52.6)	Ref	
Methods of IOL	Cook Balloon	15 (53.6)	12 (63.2)	0.673 (0.204–2.217)	0.514 b
	PGE	13 (46.4)	7 (36.8)	Ref	

Abbreviations: CI, confidence interval; IOL, induction of labor; NA, not applicable; OR, odds ratio.

a Fisher's exact test.

b Chi-square test.

**Table 3:** Factors related to the success of induction of labor following criterion 2 (vaginal birth)

Variable	Characteristics	Success ( *N* = 38)	Failed ( *N* = 9)	OR (95% CI)	*p* -Value
Maternal age (years)	<35	33 (86.8)	6 (66.7)	3.300 (0.618–17.617)	0.167 a
	≥35	5 (13.2)	3 (33.3)	Ref	
Maternal height (cm)	≥160	26 (68.4)	8 (88.9)	0.271 (0.030–2.416)	0.410 a
	<160	12 (31.6)	1 (11.1)	Ref	
Prepregnancy obesity (BMI ≥30 kg/m ^2^ )	Absent	30 (78.9)	7 (77.8)	1.071 (0.185–6.193)	1.0 a
	Present	8 (21.1)	2 (22.2)	Ref	
Obesity in pregnancy (BMI ≥30 kg/m ^2^ )	Absent	26 (68.4)	6 (66.7)	1.083 (0.231–5.081)	1.0 a
	Present	12 (31.6)	3 (33.3)	Ref	
Parity	Multiparous	14 (36.8)	4 (44.4)	0.729 (0.168–3.174)	0.716 a
	Primiparous	24 (63.2)	5 (55.6)	Ref	
Volume of amniotic fluid	Normal	31 (81.6)	6 (66.7)	2.214 (0.442–11.082)	0.285 a
	Abnormal (oligohydramnios)	7 (18.4)	3 (33.3)	Ref	
Decreased fetal movement	Absent	35 (92.1)	8 (88.9)	1.458 (0.134–15.915)	1.0 a
	Present	3 (7.9)	1 (11.1)	Ref	
Doppler abnormality	Absent	36 (94.7)	4 (44.4)	22.5 (3.240–156.269)	** 0.001 a**
	Present	2 (5.3)	5 (55.6)	Ref	
Umbilical Doppler abnormality	Absent	36 (94.7)	7 (77.8)	5.143 (0.617–42.872)	0.160 a
	Present	2 (5.3)	2 (22.2)	Ref	
CTG before IOL	Normal	37 (97.4)	7 (77.8)	10.571 (0.840–133.073)	0.090 a
	Suspected/Abnormal	1 (2.6)	2 (22.2)	Ref	
Gestational age at IOL (weeks)	≥37	36 (94.7)	4 (44.4)	22.5 (3.240–156.269)	** 0.001 a**
	<37	2 (5.3)	5 55.6)	Ref	
Newborn weight (grams)	≥2,500	13 (34.2)	1 (11.1)	4.160 (0.468–36.956)	0.244 a
	<2,500	25 (65.8)	8 (88.9)	Ref	
Bishop score before IOL (points)	≥3	22 (57.9)	3 (33.3)	2.750 (0.597–12.677)	0.270 a
	<3	16 (42.1)	6 (66.7)	Ref	
Methods of IOL	Balloon	21 (55.3)	6 (66.7)	0.618 (0.134–2.842)	0.713 a
	PGE	17 (44.7)	3 (33.3)	Ref	
Amniotomy ± oxytocin	Present	17 (44.7)	6 (66.7)	0.405 (0.088–1.862)	0.286 a
	Absent	21 (55.3)	3 (33.3)	Ref	

Abbreviations: BMI, body mass index; CTG, cardiotocography; CI, confidence interval; IOL, induction of labor; OR, odds ratio.

a Fisher's exact test.

**Fig. 2:**
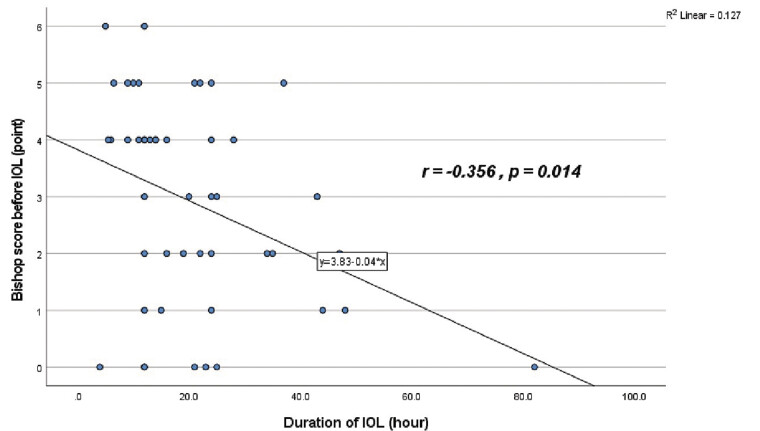
Relation between Bishop score before IOL (point) and duration of IOL (hour). IOL, induction of labor.

**Fig. 3:**
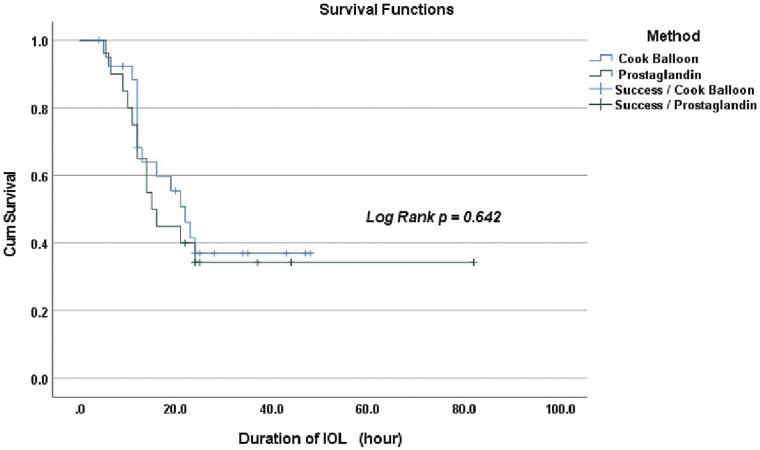
Kaplan–Meier curve presents the cum survival following the duration of IOL between 2 methods of IOL. IOL, induction of labor.

In general, the Bishop score before labor induction has a negative correlation with the time to labor induction. At the time of cervical ripening, if the Bishop score becomes more favorable, the time to labor induction will be shorter ( *r* = − 0.356, *p* < 0.05; Fig. 2 ). In addition, the success rate of IOL following duration time of IOL between Cook Balloon group and PGE group was not different significantly ( Fig. 3 ).

In this study, using univariate logistic regression, no significant factors were found to predict the success of artificial labor induction by criterion 1, with Bishop change of more than 7 points ( Table 4 ).

**Table 4:** Univariate and multivariate logistic regression in assessing factors related to the success of induction of labor following criterion 1 (Bishop score ≥7 points)

Variable	Univariate logistic regression				Multivariate logistic regression			
	B	S.E	Exp (B) (95% CI)	*p* -Value	B	S.E	Exp (B) (95% CI)	*p* -Value
Maternal age (years)	−0.041	0.061	0.960 (0.851–1.082)	0.501	NA			
Maternal height (cm)	−0.045	0.047	0.956 (0.871–1.049)	0.342	NA			
Prepregnancy obesity (BMI ≥30 kg/m ^2^ )	−0.001	0.048	0.999 (0.909–1.097)	0.978	NA			
Obesity in pregnancy (BMI ≥30 kg/m ^2^	0.014	0.050	1.014 (0.919–1.118)	0.781	NA			
Parity (multiparous)	0.486	0.624	1.625 (0.478–5.521)	0.437	NA			
Volume of amniotic fluid (normal)	0.496	0.718	1.643 (0.403–6.705)	0.489	NA			
Gestational age at IOL (≥37 weeks)	0.118	0.829	1.125 (0.221–5.714)	0.887	NA			
Newborn weight (≥2,500 g)	0.734	0.687	2.083 (0.542–8.011)	0.285	NA			
Bishop score before IOL (≥ 3 points)	0.393	0.597	1.481 (0.459–4.778)	0.511	NA			
Methods of IOL (PGE)	0.396	0.608	1.486 (0.451–4.893)	0.515	NA			

Abbreviations: BMI, body mass index; CI, confidence interval; IOL, induction of labor; NA, not applicable; PGE, prostaglandin E.

Regardless of criterion 2, using univariate logistic regression, our study found that the absence of Doppler indices and gestational age over 37 weeks were advantageous factors in predicting vaginal delivery ( *p* < 0.05). It means that more premature newborns with Doppler abnormalities were at higher risk of cesarean delivery. However, after adjusting for gestational age at IOL and newborn weight by multivariate logistic regression, no significant difference was observed ( *p* > 0.05; Table 5 ).

**Table 5:** Univariate and multivariate logistic regression in assessing factors related to the success of induction of labor following criterion 2 (vaginal birth)

Variable	Univariate logistic regression				Multivariate logistic regression a			
	B	S.E	Exp (B) (95% CI)	*p* -Value	B	S.E	Exp (B) (95% CI)	*p* -Value
Maternal age (years)	0.053	0.075	1.054 (0.910–1.221)	0.482	NA			
Maternal height (cm)	0.056	0.059	1.068 (0.943–1.187)	0.339	NA			
Prepregnancy obesity (BMI ≥30 kg/m ^2^ )	0.022	0.057	1.022 (0.913–1.143)	0.706	NA			
Obesity in pregnancy (BMI ≥30 kg/m ^2^	0.020	0.061	1.021 (0.906–1.150)	0.737	NA			
Parity (multiparous)	0.316	0.750	1.371 (0.315–5.969)	0.674	NA			
Volume of amniotic fluid (normal)	0.795	0.822	2.214 (0.442–11.082)	0.333	NA			
Decreased fetal movement (absent)	0.377	1.219	1.458 (0.134–15.915)	0.757	NA			
Method of IOL (PGE)	0.482	0.779	1.619 (0.352–7.450)	0.536	NA			
Doppler abnormality (absent)	3.114	0.989	22.500 (3.240–156.269)	**0.002**	0.966	1.445	2.628 (0.162–42.583)	0.497
Umbilical Doppler abnormality (absent)	1.638	1.082	5.143 (0.617–42.872)	0.130	NA			
CTG before IOL (normal)	2.358	1.292	10.571 (0.840–133.073)	0.068	NA			
Gestational age (≥37 weeks)	3.114	0.989	22.5 (3.240–156.269)	**0.002**	1.556	1.296	4.739 (0.374–60.039)	0.155
Newborn weight (≥2,500 g)	1.426	1.114	4.160 (0.468–36.956)	0.201	NA			
Bishop score before IOL (≥3 points)	1.012	0.780	2.750 (0.597–12.677)	0.194	NA			
Amniotomy ± oxytocin (absence)	0.904	0.779	2.471 (0.537–11.368)	0.245	NA			

Abbreviations: CI, confidence interval; CTG, cardiotocography; IOL, induction of labor; NA, not applicable; PGE, prostaglandin E; SE, standard error.

Information supplemental to the model: Omnibus tests of model coefficients ( *p* = 0.001); Cox and Snell *R* ^2^ (0.291); Nagelkerke *R* ^2^ (0.466); Hosmer–Lemeshow test ( *p* = 0.133); predicted (91.5%).

Variables with *p* -value <0.05 in univariate logistic regression were included in multivariate logistic regression.

a Adjustment for gestational age at IOL and newborn weight.

## Discussion

Our study found that the Bishop score before IOL helps in reducing the duration of IOL. In FGR pregnancies with a normal Doppler ultrasound scan and gestational age at IOL greater than 37 weeks, these are good prognostic factors for vaginal delivery.

In the study by Horowitz and Feldman, of the 131 patients who underwent induction, 83% delivered vaginally. Logistic regression showed that oligohydramnios (OR: 3.98; CI: 1.35–11.76) and prostaglandin use (OR: 3.67; CI: 1.07–12.60) were significantly associated with cesarean delivery. 11 In the study by Nwabuobi et al, a total of 594 pregnancies were included. Cesarean delivery was performed in 243 (40.9%) pregnancies. Significant risk factors associated with cesarean delivery were maternal age, gestational age at delivery, and initial method of labor induction. 13 According to Pinton et al, of the 146 women included, 56 (38.4%) had cesarean deliveries. After adjustment, the factors significantly associated with the risk of cesarean were maternal age greater than 39 years (AOR = 4.33 [1.22–17.2], reference: 25–39 years), nulliparity (ORa = 3.49 [1.25–11.2]), and an abnormal fetal umbilical artery Doppler velocimetry (ORa = 3.50 [1.47–8.70]). The risk of poor neonatal condition did not differ significantly between women with vaginal and cesarean deliveries (2.3% vs. 7.3%, *p* = 0.21). 14

In-line with Farah et al, of a total of 453 study participants who had undergone IOL, 349 (77%) of them had successful IOL with a 95% CI of 73%, 81%. Favorable Bishop score (AOR = 3.45, 95% CI: 1.98, 5.99), time from the start of induction to delivery <12 hours (AOR = 4.01, 95% CI: 2.16, 7.450), non-reassuring fetal heart rate pattern (AOR = 0.42, 95% CI: 0.22, 0.78), and amniotic fluid change to meconium (AOR = 0.43, 95% CI: 0.23, 0.79) were significantly associated with the success of labor induction. 15 In accordance with Metrop et al, among the 320 patients, 246 were delivered vaginally (76.9%), and 74 had a CS (23.1%). Prognostic factors for successful FGR induction were non-scarring uterus (OR: 8.41; 95% CI: [2.92–24.21]), absence of preeclampsia (OR: 7.14; 95% CI: [2.42–21.03]), multiparity (OR: 4.32; 95% CI: [1.83–10.18]), normal fetal heart rate before IOL (OR: 2.99; 95% CI: [1.24–7.22]), and BMI <30 (OR: 3.54; 95% CI: [1.62–7.72]). Doppler abnormalities, method and number of lines of IOL, and cervical evaluation were not significant in our study. 16

However, the predictive accuracy of these methods can be limited. Management of FGR involves careful fetal surveillance, serial fetal growth and amniotic fluid volume assessments, and determining the appropriate timing for delivery to balance the risks of stillbirth and prematurity. 1

## Conclusion

In conclusion, normal Doppler and gestation age at IOL may be advantageous factors supported for vaginal delivery among pregnancies complicated by FGR. All stakeholders should note these factors in counselling and monitoring the IOL. More studies with a larger number of patients are required to strengthen these findings.

## References

[1] Chew L C Osuchukwu O O Reed D J Verma R P Fetal Growth RestrictionStatPearls. Treasure Island (FL): StatPearls Publishing;2025. Accessed December 30, 2025 at:https://www.ncbi.nlm.nih.gov/books/NBK562268/32965939

[2] Walter A Calite E Berg C Gembruch U Müller A Geipel A Prenatal diagnosis of fetal growth restriction with polyhydramnios, etiology and impact on postnatal outcome Sci Rep 2022 12 01 415 35013541 10.1038/s41598-021-04371-9 PMC8748543

[3] Melamed N Baschat A Yinon Y FIGO (International Federation of Gynecology and Obstetrics) initiative on fetal growth: best practice advice for screening, diagnosis, and management of fetal growth restriction Int J Gynaecol Obstet 2021 152(Suppl 1):3 57 33740264 10.1002/ijgo.13522 PMC8252743

[4] Tsikouras P Antsaklis P Nikolettos K Diagnosis, prevention, and management of fetal growth restriction (FGR) J Pers Med 2024 14 07 698 39063953 10.3390/jpm14070698 PMC11278205

[5] Carlson N Ellis J Page K Dunn Amore A Phillippi J Review of evidence-based methods for successful labor induction J Midwifery Womens Health 2021 66 04 459 469 33984171 10.1111/jmwh.13238 PMC8363560

[6] Bitar G Jain V Ruhstaller K Mapp A Sciscione A Fetal growth restriction and cesarean delivery for nonreassuring fetal heart rate tracing in the late preterm period Obstet Gynecol 2020 135 117s. Accessed December 30, 2025 at:https://journals.lww.com/greenjournal/fulltext/2020/05001/fetal_growth_restriction_and_cesarean_delivery_for.410.aspx

[7] Nguyen T NTN Vuong A DB Nguyen P N Nguyen N TT Ho Q N Le Q T Using dinoprostone slow release vaginal insert for cervical ripening in term-pregnancy with oligohydramnios J Obstet Gynaecol Res 2023 49 07 1750 1761 37245054 10.1111/jog.15665

[8] Nariai M Wada-Hiraike O Iriyama T Risk factors for failed induction of labor: A retrospective study in a single, tertiary, perinatal-care center Taiwan J Obstet Gynecol 2025 64 04 687 692 40602966 10.1016/j.tjog.2025.04.010

[9] Demssie E A Deybasso H A Tulu T M Abebe D Kure M A Teji Roba K Failed induction of labor and associated factors in Adama Hospital Medical College, Oromia Regional State, Ethiopia SAGE Open Med 2022 10 20503121221081009 10.1177/20503121221081009 PMC9133872 35646365

[10] Alayu S Talie A Bishaw K A Vaginal delivery following induction and associated factors among laboring women at South Wollo Zone Public Hospitals of Ethiopia, 2023 Sci Rep 2024 14 01 25255 39448660 10.1038/s41598-024-74589-w PMC11502816

[11] Horowitz K M Feldman D Fetal growth restriction: risk factors for unplanned primary cesarean delivery J Matern-Fetal Neonatal Med 2015 28 18 2131 2134. Accessed August 19, 2025 at:https://pubmed.ncbi.nlm.nih.gov/25354283/25354283 10.3109/14767058.2014.980807

[12] Gordijn S J Beune I M Thilaganathan B Consensus definition of fetal growth restriction: A Delphi procedure Ultrasound Obstet Gynecol 2016 48 03 333 339. Accessed December 30, 2025 at:https://pubmed.ncbi.nlm.nih.gov/26909664/26909664 10.1002/uog.15884

[13] Nwabuobi C Gowda N Schmitz J Risk factors for Cesarean delivery in pregnancy with small-for-gestational-age fetus undergoing induction of labor Ultrasound Obstet Gynecol 2020 55 06 799 805 31441151 10.1002/uog.20850

[14] Pinton A Lemaire Tomzack C Merckelbagh H Goffinet F Induction of labour with unfavourable local conditions for suspected fetal growth restriction after 36 weeks of gestation: Factors associated with the risk of caesarean J Gynecol Obstet Hum Reprod 2021 50 07 101996 33217602 10.1016/j.jogoh.2020.101996

[15] Farah F Q Aynalem G L Seyoum A T Gedef G M The prevalence and associated factors of success of labor induction in Hargeisa maternity hospitals, Hargeisa Somaliland 2022: A hospital-based cross-sectional study BMC Pregnancy Childbirth 2023 23 01 437 37312039 10.1186/s12884-023-05655-w PMC10262556

[16] Metrop M Leblanc F Cailliau E Prognostic factors for successful induction of labor in intrauterine growth restriction after 36 weeks of gestation Eur J Obstet Gynecol Reprod Biol 2022 276 213 218 35939909 10.1016/j.ejogrb.2022.07.032

